# In vitro antioxidant and cholinesterase inhibitory activities of methanolic fruit extract of *Phyllanthus acidus*

**DOI:** 10.1186/s12906-015-0930-y

**Published:** 2015-11-09

**Authors:** Md. Moniruzzaman, Md. Asaduzzaman, Md. Sarwar Hossain, Jyotirmoy Sarker, S. M. Abdur Rahman, Mamunur Rashid, Md. Mosiqur Rahman

**Affiliations:** Department of Pharmacy, Southeast University, Dhaka, 1213 Bangladesh; Department of Clinical Pharmacy and Pharmacology, Faculty of Pharmacy, University of Dhaka, Dhaka, 1000 Bangladesh

**Keywords:** Antioxidants, Oxidative stress, Free radicals, Acetylcholinesterase, Butyrylcholinesterase, Alzheimer's disease, *Phyllanthus acidus*

## Abstract

**Background:**

Alzheimer’s disease (AD) is a progressive neurodegenerative disorder clinically characterized by loss of memory and cognition. Cholinergic deficit and oxidative stress have been implicated in the pathogenesis of AD. Therefore, inhibition of acetylcholinesterase and oxidation are the two promising strategies in the development of drug for AD. *Phyllanthus acidus*, belonging to the family Euphorbiaceae, is a tree and has been used in traditional medicine to treat several pain, inflammatory and oxidative stress related disorders such as rheumatism, bronchitis, asthma, respiratory disorder, also important to promote intellect and enhance memory, thus supporting its possible anti-Alzheimer’s properties. In this study, *P. acidus* was evaluated for its cholinesterase inhibitory and antioxidant activities.

**Methods:**

In this study, we evaluated the antioxidant potential and neuroprotective activity of *P. acidus* by assessing total phenol content (FCR assay), total flavonoid content, total antioxidant capacity, *Fe*^3+^ reducing power capacity, DPPH (2, 2-diphenyl-1-picrylhydrazyl) and hydroxyl radical scavenging capacity, lipid peroxidation inhibition activity & metal chelating activity. In addition acetylcholinestrase (AChE) and butyrylcholinestrase (BChE) inhibitory activities were performed using Ellman’s method.

**Results:**

Total phenolic content and total flavonoid content of the extract were 116.98 mg of gallic acid equivalent and 168.24 mg of quercetin equivalent per gm of dried extract. The methanolic extract of *P. acidus* (MEPA) showed considerable total antioxidant activity and reducing capacity. In DPPH scavenging assay and hydroxyl radical scavenging assay, the MEPA showed 84.33 % and 77.21 % scavenging having IC_50_ of 15.62 and 59.74 μg/ml respectively. In lipid peroxidation inhibition activity MEPA showed moderate inhibition of peroxidation at all concentrations with IC_50_ value of 471.63 μg/ml and exhibited metal chelating activity with IC_50_ value 308.67 μg/ml. The MEPA exhibited inhibition of rat brain acetylcholinesterase and human blood butyrylcholinesterase in a dose dependent manner and the IC_50_ value was found to be 1009.87 μg/ml and 449.51 μg/ml respectively.

**Conclusion:**

These results of the present study reveal that MEPA has considerable amount of antioxidant activity as well as anti-acetylcholinesterase and anti-butyrylcholinesterase activity which suggest its effectiveness against Alzheimer's disease and other neurodegenerative disorders.

## Background

It is highly impossible to consider a biological life without oxygen and this valuable oxygen is metabolized and produce free radicals (FR) in human body by oxidative process having an extensive effects on human health [1, 2]. FR and its by-product reactive oxygen species (ROS) are continuously produced in human body [3, 4]. In some cases (like alcohol, exposure to chemicals, stress, tobacco and UV exposure) there is an excess production of ROS. In this condition, bodily produced antioxidants are insufficient and produce imbalance called oxidative stress [5]. Super oxide anion (O_2_^−^⋅), hydroxyl radical (⋅OH) and hydrogen peroxide (H_2_O_2_) are the primary production of ROS is regulated by an enzymatic antioxidant system and the intake of vitamins related to the daily diet [6]. Over production of ROS is responsible for oxidative damage of macromolecules such as DNA, proteins, lipids, carbohydrates etc. and this damage to DNA also may produce cancer [7, 8]. As a result our body cannot protect from ROS by the primary defense system and ROS continuously oxidize the cells producing secondary ROS that leads to the oxidative chain reactions and resulting cells destruction called oxidative damage [4]. This oxidative damage can causes various acute and chronic neurodegenerative diseases related to aging such as Alzheimer, Parkinson, osteoarthritis, atherosclerosis, myocardial infarction and age related muscular degeneration [9, 10]. This oxidative damage by FR and ROS is blocked by the Antioxidants [11]. Antioxidants are the substances which neutralize body produced FR by donating one of their own electron and preventing cellular and membrane damage. Antioxidants act by several ways such as by preventing the propagation of oxidative chain reactions, by scavenging free radicals, by regulating gene expression, by being part of the redox reaction and by preventing FR formations [12–15]. To reduce the effects of oxidation both synthetic and natural antioxidants are used [2]. BHT and BHA are highly effective synthetic antioxidants but have toxic and side effects on human [16, 17]. Nitric oxide (^.^NO), superoxide anion (O_2_^−^), hydrogen peroxide (H_2_O_2_) and hypochloride ion (OCl^−^) are the natural antioxidants produced by phagocytes as protective agents against cell infection in immune responses [18]. Carotenoids polyphenols, bioflavonoids, vitamin C (ascorbic acid), vitamin E are the major natural antioxidants reported [19].

AD is a neurological disorder associated with memory loss, cognitive dysfunction, behavioral turbulence and abnormalities in activities of daily life [20–22]. AD is frequently founded in elderly people and characterized by malfunctioning of different biochemical pathways [23]. AD has been associated with a significant decrease in the amount of acetylcholine (ACh) by breaking down of ACh [24–26]. ACh is a neurotransmitter that transmits signal in the synapse, after delivering signal ACh is hydrolyzed and given choline and acetyl group in a reaction catalyzed by the enzyme AChE and its pharmacological action is done primarily by acetylcholinesterase (AChE) and secondary by butyrylcholinesterase (BChE) [27]. Over activity of AChE and BChE enzymes are responsible for the development of different neurological disorder like AD, Parkinson’s disease etc. [24, 28]. The most successful way to get rid of this problem is “cholinergic hypothesis” and the approving drugs are working to increase the ACh level in the brain [23] that will improve cognitive function [29]. AChE inhibitors tacrine, donepezile, rivastigmine, and galanthamine are only the approved drugs for the treatment of AD although having numerous side effects [30]. The mechanism based inhibitors due to its role in the hydrolysis of the neurotransmitter Ach is an attractive target for the rational drug design and for the discovery of new drugs for AD [31].

Medicinal plants have been used from ancient to the present time for the remedy of disease of human being. Galanthamine is an anticholinesterase alkaloid isolated from snowdrop approved for the treatment of AD [32]. *P. acidus* (from the family Euphorbiaceae) plant is also one of the important plants having various medicinal properties such as antioxidants and anti-inflammatory effects. Many crude plants found having antioxidant properties and among the compounds phenolic and flavonoid attracted as significant choice for being used as antioxidants [2]. Traditionally, *P. acidus* is used in the treatment of fever, respiratory disorders, diabetes, bronchitis, inflammation, several pains etc. and also helpful to cure cough, psoriasis, sudorific, to improve eyesight and memory [33]. Methanolic extract of fruits and leaves was reported to show antimicrobial effect [34]. Petroleum ether extract of fruits was reported to show cytotoxic, antibacterial and antioxidant activities [35]. The fruits and leaves of the plant yielded promising hepatoprotective activity [36]. The methanolic fruit extract of the plant reported to show antibacterial, cytotoxic and antioxidant properties [37]. But no AD related activity of methanolic fruit extract of this plant has been done yet. Thus, our main objective of the present study was to evaluate the antioxidant and neuroprotective potential of *P. acidus* to treat the AD and other neurodegenerative diseases.

## Methods

### List of chemicals

Folin–Ciocalteu reagent, Methanol, Gallic acid, Ascorbic acid, DPPH, 2-deoxy-D-ribose, Thiobarbituric acid (TBA), (+)-Catechin, 5,5´-dithio-bis-(2-nitro) benzoic acid (DTNB), Acetylthiocholine iodide, S-Butyrylthiocholine iodide, Donepezil, Ferrozine monosodium,Trichloro acetic acid (TCA) and Triton X-100 were purchased from Sigma chemical company, USA. Butylated hydroxyl toluene (BHT) and Tris–HCl buffer were purchased from Merck, Germany.

### Collection of plant

The fruits of *P.acidus* were collected from Kapasia, in the district Gazipur of Bangladesh in August 2014, and identified by an expert taxonomist from the Bangladesh national herbarium. A voucher specimen (DACB, ACCESSION NUMBER:40181) was preserved in the national herbarium, Dhaka, Bangladesh for future reference.

### Preparation of extract

The collected fresh fruit of *P. acidus* weighing 5 kg was then washed properly to remove dirty materials and shade dried for several days with occasional sun drying. These were then dried in an oven for 24 h at considerably low temperature for better grinding. The grounded powder (500 g) was macerated with methanol (2.5 L) and extracted by cold extraction process. Finally 15 g methanol extract was obtained after evaporating the filtrate.

### Determination of phytoconstituents

#### Determination of total phenolics

Total phenolic content of *P. acidus* was determined according to the method of Singleton V. L. *et al.,* [38] with minor modifications using Folin-Ciocalteu reagent. Each test tube contained 0.5 ml of plant extract or standard solution at different concentrations, 2.5 ml of Folin–Ciocalteu reagent solution (10 times diluted with water) and 2.5 ml of Sodium carbonate (7.5 %) solution. After adding all of the reagents the test tubes were incubated for 25 min at 25 °C to complete the reaction and the absorbance of the solution was measured at 760 nm. A standard curve was prepared using gallic acid as standard (Y = 0.0151x + 0.059, R^2^ = 0.9913) for expressing the total content of phenolic compounds in plant extract and shown as mg of gallic acid equivalent (GAE)/gm of dried extractives.

#### Determination of total flavonoids

Total flavonoid content was determined by the aluminum chloride colorimetric method described by Barrera et al., [35], and quercetin was used as standard. Briefly, 1.0 ml of plant extract or standard of different concentration were added to 3 ml of methanol, 0.2 ml of 10 % AlCl_3_, 0.2 ml of 1 M potassium acetate and 5.6 ml of distilled water. After incubation for 25 min the absorbance was taken at 420 nm. A quercetin standard curve was prepared (Y = 0.009x + 0.036, R^2^ = 0.972) to express the result as mg of quercetin equivalent(QE)/g of dried extractives.

### Antioxidant ability assay

#### Determination of total antioxidant capacity

Total antioxidant capacity was determined according to the method as described by Prieto P. et al., [39] with some modifications. In this experiment, 0.5 ml of MEPA or standard (ascorbic acid) of different concentration (100 – 600 μg/ml) was added to 3 ml of reaction mixture (containing 0.6 M sulphuric acid, 28 mM sodium phosphate and 1 % ammonium molybdate) into the test tube. After incubating for15 min at 90°C for completing the reaction followed by cooling at room temperature the absorbance was measured at 695 nm. Ascorbic acid (AA) was used as standard in this study.

#### Reducing power capacity assessment

The reducing power was evaluated by the method of Oyaizu [37]. In this method, various concentrations of MEPA or standard solutions (1.0 ml) were mixed with 2.5 ml of potassium buffer (0.2 M, pH 6.6) and 2.5 ml of Potassium ferricyanide [K_3_Fe (CN) _6_] (1 %) solution. After 30 min incubation at 50^0^ C, 2.5 ml of trichloro acetic acid (10 %) solution was added into the test tube. The total mixture was centrifuged at 3000 *g* for 10 min. Then 2.5 ml supernatant solution was withdrawn from the mixture and mixed with 2.5 ml of distilled water and 0.5 ml of FeCl_3_ (0.1 %) solution. Then the absorbance of the solution was measured at 700 nm and AA was used as standard.

#### Determination of DPPH radical scavenging activity

DPPH radical scavenging activity was determined according to the method as described by Choi et al., [40]. 2 ml of methanolic solution of plant extract or standard (BHT) at different concentration was mixed with 3 ml (0.02 %) of methanol solution of DPPH. After incubation for 30 min at dark place the absorbance was taken at 517 nm against methanol as blank.

#### Determination of hydroxyl radical scavenging assay

Hydroxyl radical scavenging activity of different concentrations of MEPA was determined by the method of Elizabeth et al., [41]. Hydroxyl radical was generated by the *Fe*^3+^-ascorbate-EDTA-H_2_O_2_ system (the Fenton reaction). 1 ml of reaction mixture was made by adding 2-deoxy-D-ribose (2.8 mM), KH_2_PO_4_-KOH buffer (20 mM, pH 7.4), FeCl_3_ (100 μM), EDTA (100 μM), H_2_O_2_ (1.0 mM), AA (100 μM) and various concentrations of the test sample or reference compound [(+)- catechin)]. After incubation for 1 h at 37 °C, 0.5 ml of the reaction mixture was mixed with 1 ml of 2.8 % TCA and 1 ml of 1 % aqueous TBA then the mixture incubated at 90 °C for 15 min to develop the color. After cooling, the mixture’s absorbance was measured at 532 nm against an appropriate blank solution.

#### Determination of metal chelating activity assay

The chelating activity of MEPA for ferrous ion (Fe^2+^) was measured according to the method of J. Sabate [42], using ferrozine (substrate) and ferrous chloride (FeCl_2_). In this method, 0.5 ml of extract or standard was added to 1.6 ml of FeCl_2_(2mM). After incubation for 30 s, 0.1 ml ferrozine (5 mM) was added and kept 10 min at room temperature then the absorbance of the Fe^2+^ –Ferrozine complex was measured at 562 nm. A typical blank solution contained all reagents except plant extract or standard (BHT) solution.

#### Determination of lipid peroxidation inhibition activity

The inhibition of lipid peroxidation activity was evaluated according to the method as described by Liu et al., [43], with a slight modification. The adult long Evan rats weighing 150 g were anesthetized with sodium phenobarbitone. The brain of rats were dissected and homogenized with a homogenizer in ice-cold Phosphate buffer (50 mM, pH 7.4) to produce a 1/10 homogenate. The homogenate was centrifuged at 10,000 *g* for 20 min at 4 °C. The supernatant was used as liposome for in vitro lipid peroxidation assay. The ability of MEPA to inhibit lipid peroxidation was studied by incubating rat brain homogenates treated with hydrogen peroxide (10 μM) and different concentrations of extract or standard solution. Hydrogen peroxide induced lipid peroxidation in rat brain homogenates. 1 ml of 0.15 M KCl and 0.5 ml of liposome containing brain homogenate were added with different concentrations of plant extract or standard solution. The reaction was started by adding 100 μl of 0.2 mM ferric chloride with the above mentioned mixture then incubated at 37 ° C for 30 min. The reaction was stopped by adding 2 ml of 0.25 N HCl, 15 % TCA, 0.5 % BHT and 0.38 % TBA solution. Lipid peroxides reacted with TBA to form a pink product, thiobarbituric acid reacting substances (TBARS), measurable colorimetrically at 532 nm. The difference between the control and the test sample is the measurement of decrease in TBARS formation, reflecting reduced hydroxyl radical induced lipid peroxidation. (+)-Catechin was used as standard for comparison.

### Determination of AChE inhibitory activity

The AChE inhibitory activity was performed according to the colorimetric method of Ellman’s et al., [40, 44] using acetylthiocholine iodide as a substrate. For the enzyme source, the rat brains were homogenized in a homogenizer with 5 volumes of a homogenization buffer [10 mMTris-HCl (pH 7.2), which contained 1 M NaCl, 50 mM MgCl_2_ and 1 % Triton X-100], and centrifuged at 10,000 *g* for 30 min. The resulting supernatant was used as an enzyme source. All of the extraction steps were carried out at 4 °C. Protein concentration was determined using the BCA kit (bicinchoninic acid; Sigma Co., USA) with bovine serum albumin (BSA) as a protein standard. The rates of hydrolysis by AChE were monitored spectrophotometrically. Each MEPA or standard solution (500 μl) was mixed with an enzyme solution (500 μl). After incubation at 37 °C for 15 min the absorbance was measured at 405 nm immediately after adding an Ellman’s reaction mixture (3.5 ml; 0.5 mM acetylthiocholine iodide, 1 mM DTNB) in a 50 mM sodium phosphate buffer (pH 8.0) to the above reaction mixture. Reading was repeated for 10 min at 2 min intervals to verify that the reaction occurred linearly. The blank reaction was measured by substituting saline for the enzyme. Donepezil was used as standard.

### Determination of BChE inhibitory activity

The BChE assay was performed according to the colorimetric method of Ellman’s *et al.,* [40, 44], with some modifications using s-butyrylthiocholine iodide as a substrate. For the enzyme source, the human blood was homogenized in a homogenizer with 5 volumes of a homogenization buffer [10 mM Tris–HCl (pH 7.2), which contained 1 M NaCl, 50 mM MgCl_2_ and 1 % Triton X-100], and centrifuged at 10,000 *g* for 30 min. The resulting supernatant was used as an enzyme source. All of the extraction steps were carried out at 4 °C. The rates of hydrolysis by BChE were monitored spectrophotometrically. Each MEPA or standard solution (500 μl) was mixed with an enzyme solution (50 μl) and incubated at 37 °C for 15 min. Absorbance at 405 nm was read immediately after adding an Ellman’s reaction mixture (3.5 ml; 0.5 mM S-butyrylthiocholine iodide, 1 mM DTNB) in a 50 mM sodium phosphate buffer (pH 8.0) to the above reaction mixture. Reading was repeated for 10 min at 2 min intervals to verify that the reaction occurred linearly. The blank reaction was measured by substituting saline for the enzyme. Donepezil was used as standard.

### Calculations and statistical analysis

The percentage inhibitions or scavenging of DPPH radicals, hydroxyl radicals, metal chelating, lipid peroxidation, AChE and BChE inhibitory activity of the MEPA were calculated by using the formula:

$$ \mathrm{Percentage}\ \mathrm{inhibition}\ \mathrm{or}\ \mathrm{scavenging} = \left\{\left({\mathrm{A}}_{\mathrm{o}}\hbox{--}\ {\mathrm{A}}_1\right)/{\mathrm{A}}_{\mathrm{o}}\right\}\times 100 $$

Where,

A_0_ is the absorbance of the control, and

A_1_ is the absorbance of the extract/standard.

The IC_50_value (the concentration of the extract required to scavenge 50 % of radicals or to inhibit 50 % of enzyme activity) was calculated for the standard and MEPA. The IC_50_ values of different studies shown in Table 1.

**Table 1:** IC_50_ values obtained in the radical scavenging and enzyme inhibitory activity assays

Sample	IC_50_ (μg/ml)					
	DPPH scavenging	Hydroxyl radical scavenging	Metal chelating	Inhibition of lipid peroxidation	AChE	BChE
BHT	3.48 ± 0.17	-	16.21 ± 0.18	-	-	-
(+)-Catechin	-	15.20 ± 0.38	-	58.20 ± 1.09	-	-
Donepezil	-	-	-	-	31.83 ± 0.49	16.54 ± 0.21
MEPA	15.62 ± 0.32	59.74 ± 1.57	308.67 ± 6.40	471.63 ± 15.23	1009.87 ± 19.27	499.51 ± 7.42

Each value is expressed as a mean ± standard deviation (*n* = 3)

Statistical analyses were carried out in triplicate. All results are expressed as mean ± standard deviation (SD) values average from 3 independent experiments. Free R-software version 2.15.1 (https://cran.r-project.org/bin/windows/base/old/2.15.1/) and Microsoft Excel 2007 (Roselle, IL, USA) were used for the statistical and graphical evaluations.

## Results and discussions

### Determination of phytoconstituents

#### Total phenolics

Due to presence of hydroxyl group phenolic compounds possess primary antioxidant property with free radical neutralizing activity [45]. Total phenolic content of MEPA was determined using Folin-Ciocalteu reagent. Total phenolic content of the sample was calculated on the basis of the standard curve for gallic acid and the results were expressed as mg of gallic acid equivalent (GAE)/gm of dried extractives. The phenolic content of MEPA was 58.12 mg of GAE / gm of dried extract. In a previous study the phenolic content of methanolic fruit extract of *Phyllanthus emblica* L. (Family- Euphorbiaceae) was found to be 120.9 mg of GAE / gm of dried extract which is higher in amount than MEPA [46]. According to epidemiological studies prolonged use of polyphenol enriched diet can provide protection from many chronic diseases including AD [47].

#### Total flavonoids

Flavonoids are known as reputed antioxidants due to their radical scavenging, metal ion chelating and lipid peroxidation inhibiting activities [48, 49]. Flavonoids may prevent or slow the progression of AD by interfering with the generation and polymerization of amyloid-β peptides into neurotoxic oligomeric aggregates and also by reducing aggregation of tau proteins [50]. Total flavonoids content of MEPA was determined using much known aluminum chloride colorimetric method. Flavonoid content of the samples was calculated on the basis of the standard curve for quercetin and the results were expressed as mg of quercetin equivalent (QE)/g of dried extractives. The flavonoid content of MEPA was 168.24 mg of QE/g of dried extract which is very high in comparison with the flavonoid content of methanolic fruit extract of *Phyllanthus emblica* L. reported earlier [46].

### Antioxidant activity

#### Total antioxidant capacity

**Fig. 1:**
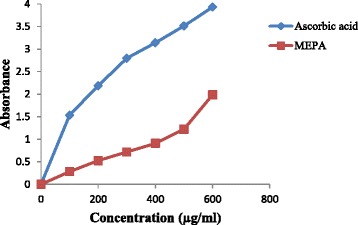
Total antioxidant activity of the MEPA and ascorbic acid at different concentrations

Total antioxidant capacity can be useful to determine nutritional interventions with antioxidant enriched foods on risk and prevention of AD [51]. Total antioxidant capacity was assessed by phosphor-molybdenum method. The phosphor-molybdenum method was based on the reduction of Mo (V1) to Mo (v) by the antioxidant compound and the formation of green phosphate/ Mo (v) complex with a maximal absorption at 695 nm. The MEPA showed considerable antioxidant activity compared to ascorbic acid (AA). At the concentration 100 μg/ml, the absorbance of MEPA and AA were 0.283 and 1.535 respectively. The extract was found to increase the total antioxidant activity with the increasing concentration of the extract. At 600 μg/ml concentration, the absorbance of MEPA and AA were 1.986 and 3.930 respectively depicted in Fig. 1. Total antioxidant capacity of methanol and water extracts of *Phyllanthus niruri* and *Phyllanthus urinaria* leaves were performed in a previous study showing that MEPA has less activity than two extracts of these plants [52].

#### Reducing power capacity

**Fig. 2:**
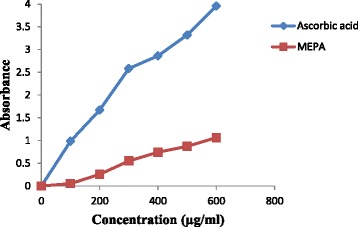
Reducing power capacity of the MEPA and ascorbic acid at different concentrations

The reducing capacity of a compound is a significant indicator of its potential antioxidant activity because of its ability to break the free radical chain through donation of a hydrogen atom. The Fe^3+^ reducing power of MEPA was determined by the method of Oyaizu [37]. The MEPA showed reducing activity less than that of AA which is a reference antioxidant. The extract showed increased reducing activity with the increasing concentration of the extract. At concentration 100–600 μg/ml the absorbance of MEPA and AA were 0.045 - 0.872 and 0.984 - 3.950 respectively which shown in Fig. 2. In an earlier study the reducing power capacity of methanolic fruit extract of *Phyllanthus emblica* L. was performed and the result is quite similar like MEPA which demonstrate that the MEPA has considerable iron reducing capacity [46].

#### DPPH radical scavenging activity

**Fig. 3:**
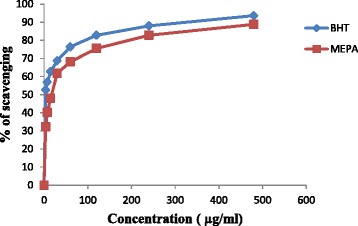
DPPH radical scavenging activity of MEPA and BHT at different concentrations

**Fig. 4:**
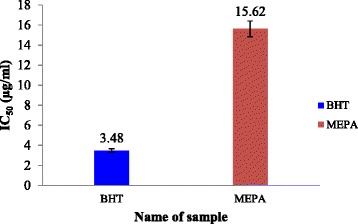
IC_50_ (μg/ml) values of BHT and MEPA for DPPH radical scavenging activity

Radical scavenging activities are very important to prevent the deleterious role of free radicals in AD. The model of scavenging the stable DPPH radical is a widely used method to evaluate the free radical scavenging ability of various samples including plant extracts [53]. DPPH antioxidant assay is based on the ability of DPPH, a stable free radical, to decolorize in the presence of antioxidants. The DPPH radical contains an odd electron, which is responsible for the absorbance at 517 nm and also for a visible deep purple color. When DPPH accepts an electron donated by an antioxidant compound, the DPPH is decolorized, which can be quantitatively measured from the change in absorbance and % of scavenging activity is calculated. The activity was increased by increasing the concentration of the sample extract. The antioxidant activity of the extractive of *P. acidus* was evaluated by DPPH radical scavenging activity. The results of DPPH radical scavenging activity of MEPA and butylated-hydroxyl-toluene (BHT) (standard) are shown in Fig. 3, and the scavenging activity of the MEPA was less than that of BHT. The IC_50_ of BHT and MEPA were 3.48 μg/ml and 15.62 μg/ml respectively shown in Fig. 4. In comparison with a former published work [46], the IC_50_ value of MEPA is about equal in value to the earlier report and it is very close to that of standard BHT. These results suggest that MEPA has high radical scavenging activity.

#### Hydroxyl radical scavenging activity

**Fig. 5:**
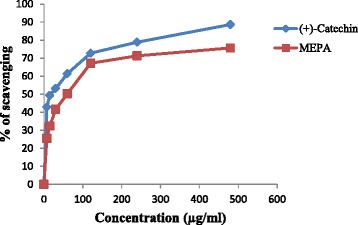
Hydroxyl radical scavenging activity of MEPA and (+)-catechin at different concentrations

**Fig. 6:**
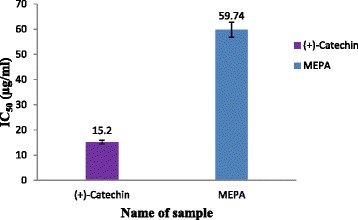
IC_50_ (μg/ml) values of (+)-catechin and MEPA for Hydroxyl radical scavenging activity

Hydroxyl radicals are the major reactive oxygen species causing lipid oxidation and enormous biological damage [43]. By incubating Fe^3+^-EDTA with H_2_O_2_ and ascorbic acid at pH 7.4, hydroxyl radicals can be generated in free solution and detected by their ability to degrade 2-deoxy-2-ribose into fragments that on heating with TBA at low pH forms a pink chromogen [43, 54]. In the hydroxyl radical scavenging activity the ability of *P. acidus* to remove the formed hydroxyl radical in solution was evaluated quantitatively by colorimetric method as included in materials and methods. When plant extractive and the reference compound, (+)-catechin, added to the reaction mixture they removed hydroxyl radicals from the sugar and prevented degradation. Removal of hydroxyl radicals from the reaction mixture decolorized the pink chromogen which can be quantitatively measured from the change in absorbance at 532 nm. The results demonstrate that the MEPA possesses hydroxyl radical scavenging activity in comparison with the standard (+)-catechin and the activity increased by increasing the concentration of the plant extractive which shown in Fig. 5. IC_50_ value of MEPA and (+)-catechin were 59.74 μg/ml and 15.20 μg/ml respectively shown in Fig. 6. The hydroxyl radical scavenging activity of methanolic fruit extract of *Phyllanthus emblica* L. was performed in a previous study and the result demonstrated that MEPA has lower hydroxyl radical scavenging activity than *Phyllanthus emblica* L [46].

#### Metal chelating activity

**Fig. 7:**
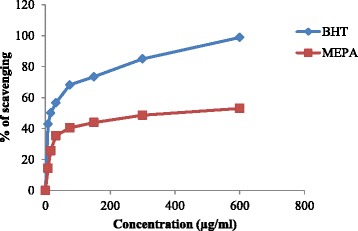
Metal chelating activity of MEPA and BHT at different concentrations

**Fig. 8:**
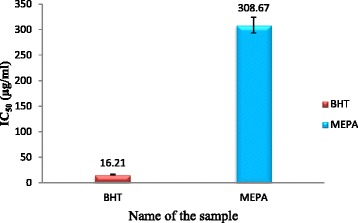
IC_50_ (μg/ml) values of BHT and MEPA for Metal chelating activity

Free radical reactions can be catalyzed by Fe^2+^ which may lead to oxidative damage of brain found in AD patients. Therefore, substances having metal chelating activity may be used to delay the rate of progression and onset of AD [55]. The chelating activity of MEPA for ferrous ions was measured according to the method as described by J. Sabate [42]. In this method free iron binds in the blood stream and enhancing metal elimination in the urine which reduces the damage done to various organs and tissues, such as the liver and nerves and estimates chelating using Ferrozine (substrate) and ferrous chloride (FeCl_2_). The inhibitory activity of MEPA increased with increasing concentration and the highest activity (53.03 %) was obtained at 600 μg/ml concentration but at the same concentration BHT (Standard) gave 98.84 % inhibitory activity that shown in Fig. 7. The IC_50_ value of BHT and MEPA were 16.21 μg/ml and 308.67 μg/ml respectively shown in Fig. 8. In comparison with a previous report on metal chelating activity of methanolic fruit extract of *Phyllanthus emblica* L. and with standard BHT, MEPA demonstrated lower ferrous ion chelating activity [46].

#### Lipid peroxidation inhibition activity

**Fig. 9:**
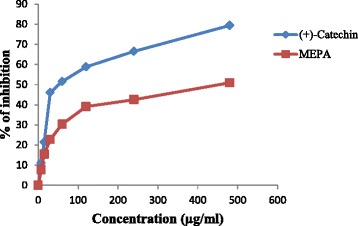
Lipid peroxidation inhibition activity of MEPA and (+)-catechin at different concentrations

**Fig. 10:**
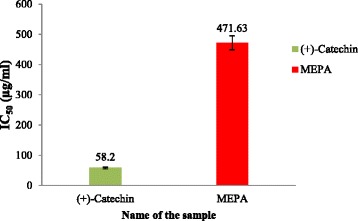
IC_50_ (μg/ml) values of (+)-catechin and MEPA for Lipid peroxidation inhibition activity

Reactive oxygen species produced by ultraviolet light, ionizing radiation, chemical reactions, and metabolic processes have numerous pathological effects, such as causing lipid peroxidation, protein peroxidation, DNA damage, and cellular degeneration related to a variety of diseases including Alzheimer’s disease [54, 56]. Lipid peroxidation has been reported to be elevated in the brain of AD. During lipid peroxidation, low molecular weight end products, generally malonaldehyde, are formed by oxidation of poly-unsaturated fatty acids that may react with two molecules of thiobarbituric acid to give a pinkish red chromogen [57]. In the lipid peroxidation inhibition activity, the activity of MEPA against non-enzymatic lipid peroxidation in rat brain homogenate was evaluated. Addition of Fe^2+^- ascorbate to the brain homogenate caused an increase in lipid peroxidation which can be quantitatively measured from the change in absorbance at 532 nm and % of inhibition activity of different concentrations were shown in Fig. 9. The IC_50_ value of (+)-catechin and MEPA were 58.20 μg/ml and 471.63 μg/ml respectively shown in Fig. 10. The lipid peroxidation inhibition activity of methanolic fruit extract of *Phyllanthus emblica* L. was performed in an earlier study and the results show that MEPA has considerable inhibitory activity against lipid peroxidation [46].

### AChE inhibitory activity

**Fig. 11:**
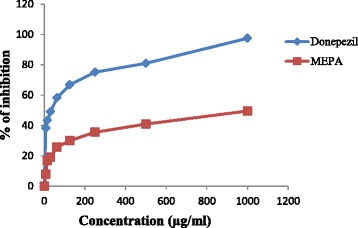
AChE inhibitory activity of MEPA and Donepezil at different concentrations

**Fig. 12:**
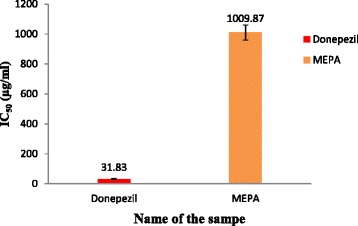
IC_50_ (μg/ml) values of Donepezil and MEPA for AChE inhibitory activity

Preventing breaking down of ACh is responsible for the elevation of ACh level in the synaptic cleft by inhibition of AChE is the most significant changes observed in AD [42]. The inhibitory activity of MEPA against rat brain AChE was determined by Ellman’s method [44]. This method estimates the level of AChE using acetylthiocholine iodide (substrate) and DTNB. The enzymatic activity was measured by the yellow color compound produced by thiocholine when it reacts with dithiobisnitro benzoate ion. The inhibitory activity of MEPA increased with increasing concentration and the highest activity was obtained at 1000 μg/ml which is 49.52 % but, at the same conditions donepezil gave 97.51 % inhibitory activity shown in Fig. 11. The IC_50_ value of donepezil and MEPA were 31.83 ± 0.49 μg/ml and 1009.87 ± 19.27 μg/ml respectively shown in Fig. 12. The AChE inhibitory activity of aqueous extract of *Emblica officinalis* (Family: Euphorbiaceae) was reported in an earlier study [58]. The comparison between IC_50_ values of aqueous extract of *Emblica officinalis* and MEPA demonstrates that it has lower acetylcholinesterase inhibitory activity than *Emblica officinalis* [58].

### BChE inhibitory activity

**Fig. 13:**
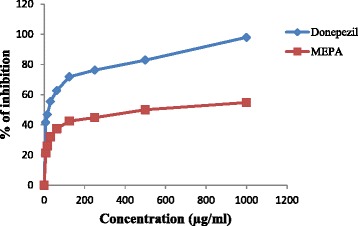
BChE inhibitory activity of MEPA and Donepezil at different concentrations

**Fig. 14:**
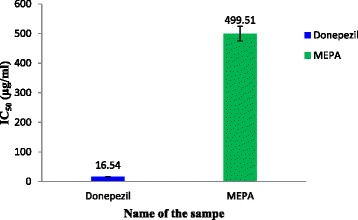
IC_50_ (μg/ml) values of Donepezil and MEPA for BChE inhibitory activity

The inhibitory activity of MEPA against human blood BChE was determined by Ellman’s method [44] with some modifications. This method estimates BChE using s-butyrylythiocholine iodide (substrate) and DTNB. The enzymatic activity was measured by the yellow color compound produced by thiocholine when it reacts with dithiobisnitro benzoate ion. The inhibitory activity of MEPA increased with increasing concentration and the highest activity was obtained at 1000 μg/ml which is 54.78 % but, at the same conditions donepezil gave 97.92 % inhibitory activity that shown in Fig. 13. The IC_50_ value of donepezil (Standard) and MEPA were 16.72 ± 0.21 μg/ml and 499.51 ± 7.42 μg/ml respectively shown in Fig. 14. The comparison between IC_50_ values of aqueous extract of *Emblica officinalis* and MEPA demonstrates that it has lower BChE inhibitory activity than *Emblica officinalis* [58].

## Conclusion

This present study gathered experimental evidence that the methanol extract obtained from fruits of *P. acidus* presented substantial amount of polyphenols and flavonoids and exhibited potential antioxidants and radical scavenging activities which are comparable with the standard (references) drugs by scavenging various free radicals as well as effectively inhibits AChE and BChE activity. Therefore, the plant has promising compounds to be tested as potential drugs for the treatment of diseases resulting from oxidative stress like AD. Due to the presence of significant antioxidant activity of this plant, further studies are underway for isolation and identification of lead compound(s) to prevent the AD and other neurodegenerative disorders.
